# Editorial

**Published:** 1868-02

**Authors:** 


### Editorial.

Medical Education.—Nearly nine months have elapsed since the Convention of Delegates from a large part of the Medical Colleges in the country was held in Cincinnati, and, after full and free discussion, adopted, with much unanimity, certain propositions relating to important changes in the mode and extent of medical college instruction. The propositions adopted, with the record of proceedings of the Convention, were extensively published in the medical periodicals of the country, and several months have elapsed since a Committee, appointed by that Convention, presented in due form a copy of the same propositions to the Faculties of each of the colleges in the United States, with a request that explicit action should be taken on the question of their approval and practical adoption, or amendment, or rejection. What responses have been made by the several colleges to the Committee it is not our purpose at present to state. It is exceedingly desirable, however, that all the *Faculties* who have not responded should do so before the first of March next, that the Committee may be enabled to decide whether it is expedient or necessary to call another convention or not.

The propositions submitted for the consideration and adoption of the colleges, have elicited more or less discussion in all the important medical periodicals of the country, and have received a cordial and almost unanimous approval. In regard to the necessity of exacting a standard of preliminary education there is not a dissenting voice. One or two have objected to the proposed extension of the period of study to *four* years; and one (*Cincinnati Lancet and Observer*), while admitting the desirableness and great advantages to result from the propositions to require attendance on three courses of lectures, and to give the instruction in the several branches in consecutive series, instead of heterogeneous, advocates delay in their actual adoption in practice, on the singular ground that the *profession* are not prepared to sustain them at this time. The editor expresses the hope that the time may come when the *profession* will be *educated up* to the point of affording the necessary support. To talk of *educating the profession at large up* to the point of sustaining the measures proposed by the late Convention at Cincinnati sounds queerly at this day, and makes one almost suspect that our neighbor of the *Lancet* has just awakened from a Rip Van Winkle sleep on this subject. Why, neighbor, the profession at large was *educated up* on this subject more than twenty years since, and gave full proof of the fact in the adoption of propositions having the same end in view at the first meeting of the American Medical Association in 1847. And the profession at large, through their commissioned representatives in State and National Associations, have been almost annually reiterating the demand on the colleges for systematic and thorough improvements in our system of medical college instruction.

Our friend simply put the boot on the wrong foot. It is not the *profession at large* that needs "educating up," but the Faculties of the several colleges. And, even here, it is not an education of the intellect that is needed. For we do not believe there are ten professors in all the regular medical colleges in the United States, who could look each other in the face and seriously claim that the changes proposed, in all their essential features, were not correct and of great practical importance. It is rather in the line of mutual confidence in the integrity of each other, and in a little of that independent professional patriotism that practically places the interests of the profession and the community above the temporary interests of *self*, that additional education is needed. There is no longer any propriety in "whipping the Devil around the bush" in regard to improvements in our system (or rather want of system) of medical education. The real obstacle in the way of any and all desirable improvements is a puerile distrust of each other, entertained by the faculties of the several schools.

A quarter of a century ago, when this subject was under discussion in the Medical Society of the State of New York, the delegates from the schools of that State opposed every proposition for increasing the extent of instruction or requirements for graduation, on the ground that the standard in these respects was as high in New York as in any of the surrounding States; and if their schools exacted more it would only drive students from them to those of New England and Pennsylvania. It is precisely the same to-day. The schools of New York hesitate to take a single decidedly advanced step, through fear that those of Boston and Philadelphia will not in "*good faith*" do the same; and those of Philadelphia are influenced by the same distrust of those in New York. The school at Albany hesitates through distrust of those at Pittsfield, Woodstock, and Buffalo. Those of Buffalo and Chicago, most of them, stand shivering behind the *free* State dignity of the Michigan University, while the latter wastes its energies in fighting off homœopathy and other *isms* with the State Legislature. Those of Cincinnati and St. Louis distrust those of Louisville, Nashville, and New Orleans, and the latter reciprocate the same.

It was expressly to obviate this distrust, by affording a medium through which concert of action might be procured, that the American Medical Association was originally called into existence. The Faculties of the several colleges, feeling the force of general professional sentiment, as strongly and repeatedly expressed through that and State organizations, and conscious of the correctness of the demands upon them for improvements, have still allowed this selfish distrust to hold sway, and, with two or three exceptions, have, all these twenty years, endeavored to parry the just demands of the profession and the permanent interests of humanity, by such temporizing expedients as two or three weeks of preliminary lectures before the commencement of the regular annual lecture terms, or the addition of summer courses on special topics, all of which make a show on paper, but are of little real importance, simply because they are never attended by one-fifth of the whole number of students. The action of the recent Convention at Cincinnati has afforded an opportunity for the colleges to abandon all these expedients and come directly, in full concert, up to the reasonable requirements of the profession.

How far the several faculties can overcome their mutual distrust, and foolish ambition for *large* classes instead of *well-qualified* ones, their responses to the Committee of the Convention will show. Let the questions proposed be answered with a directness and candor becoming the dignity and interests of an honorable and learned profession. If they do not show sufficient harmony in regard to all the details of the present plan, let another convention be called to complete the adjustment of differences at once. We believe the time has fully come, when temporizing expedients and excuses will no longer satisfy the great mass of the profession. The existing medical schools must either adopt, in good faith, substantially, the improvements proposed by the recent Convention, or see new schools, organized on the basis indicated, spring into existence in every large city in the whole country. As the new schools thus forced into existence, by the non-action of the present ones, will require of the student a longer period of study, attendance on more courses of lectures, better preliminary qualifications, and annual examinations as they progress from one series to the next, they must and will endeavor to offset these increased demands upon the time and attainments of the student, by lowering the rate of lecture fees. It needs no prophetic inspiration, to see the alternatives and tendencies here indicated; for they are logically deducible from the present relations of the schools to the profession. The Faculties connected with the colleges in most of the larger cities have been, and still are, anxious to secure uniformity of action in favor of a reasonably high standard of lecture fees. We respectfully suggest to them that the speediest way to accomplish permanently such a result, is to adopt in full the recommendations of the Cincinnati Convention, which would result in rapidly concentrating all medical college instruction in such populous centres as would furnish the required hospitals for the clinical courses; and would thereby remove nearly all the obstacles in the way of adopting a uniform and fair standard of *fees*. It is said that a word to the wise is sufficient.

Chicago Medical Society.—At a recent meeting of this Society, Dr. E. L. Holmes presented a section of the œsophagus, from the interior lining of which had been developed a morbid growth or tumor presenting all the microscopic and other characteristics of medullary cancer. The chief point of interest in the history of the case, was the very short period of time that the patient had suffered any inconvenience in swallowing food or drinks. The whole duration of his sickness was reported to have been only six weeks; and the first two of these he complained but little.

The remainder of the meeting was occupied with a discussion concerning the *modus operandi* and therapeutic application of aconite, digitalis, and veratrum viride. Dr. N. S. Davis, in opening the discussion, stated briefly the experiments and observations that had been made for determining the special action of digitalis and veratrum viride and deduced therefrom, as well as from observation of their effects in ordinary practice, the following conclusions:

1st. Digitalis acts upon the organic nervous and muscular systems in such a manner as to increase the contractility of the latter, and thereby increase the force of the contractions of the heart, the muscular fibres of the gravid uterus, etc.; while, secondarily, it increases the secretion from the kidneys, and, in large doses, often excites purging with abdominal pains and bloody stools.

2d. Veratrum viride acts as a direct sedative, diminishing both the force and frequency of the action of the heart and arteries, and, in full doses, inducing free emesis. Unlike digitalis, it relaxes the tone and impairs the contractility of the muscular structures. Whether it produces its sedative action by a primary influence on the organic nervous system, or by a direct modification of the elementary susceptibility of the tissues, is not positively determined.

3d. The veratrum viride, as a therapeutic agent, may be used with advantage in all those diseases characterized by frequency and fulness or tension of the circulation; but not when frequency is associated with positive weakness of pulse or muscular relaxation. On the contrary, digitalis is applicable, as a sedative, to such cases only as present feebleness with frequency of cardiac and arterial action, and still more, if such feebleness is associated with a morbidly excitable condition of the nervous structures, as in certain cases of delirium tremens, hysteria, etc.

The discussion was participated in by Drs. Hatch, Paoli, H. M. Lyman, Reid, and Holmes.

Dr. Paoli spoke more particularly of the therapeutic value of aconite, which he regards as both sedative and narcotic; and, consequently, capable of application to the treatment of almost all diseases of excitement. He prefers the tincture or fluid extract of the root, which he gives in doses of half a minim to a minim, repeated every one or two hours until the desired effect is induced, then lengthening the interval, to avoid accumulation of action. He regards it as necessary to watch the effects of the remedy closely while administering it as a sedative. He regarded digitalis as applicable and sometimes promptly beneficial in cases of feebleness and frequency of cardiac action, but positively dangerous in *hypertrophy* and active irritation.

Dr. Lyman had not used digitalis as a sedative, but only as a diuretic. He regarded both veratrum viride and aconite as very valuable arterial sedatives. He had used a combination of aconite and morphia with excellent effects, as a substitute for pulv. Doveri, in procuring sleep and diaphoresis. The same combination also promptly relieves some cases of neuralgia, and is much less liable to be followed by secondary nausea and vomiting than the preparations of opium given alone. Dr. Gross had given the same combination with benefit in cases of dysmenorrhœa. He regarded the administration of aconite, as a sedative, more dangerous than veratrum viride, because the latter limits its own action by the induction of vomiting.

Dr. Reid had used aconite as a sedative more frequently than veratrum viride. His opinions and experience coincided with those of Dr. Paoli. He had seen some cases, in which numbness and prickling in the fingers was induced by taking a single dose of *one drop* of the tincture of aconite root.

---

Chicago Medical College.—The Public Commencement of the Chicago Medical College will be held in the afternoon of the first Tuesday in March, at the College, on the corner of State and Twenty-Second Streets. The valedictory address will be delivered by Prof. E. Andrews. On Monday afternoon preceding the commencement, a public meeting will be held, commencing at 2½ o'clock, at which the candidates for graduation will be publicly examined in class, and some of them required to read their theses. This meeting will be at the College, and censors and members of the State and City Medical Societies are especially invited to attend and participate in the examinations. On Tuesday, a meeting of the Alumni Association of the College will be held, for the renewal of acquaintances, reading of papers, and the transaction of business. After the close of the public commencement exercises, on Tuesday evening a social entertainment will be given to the graduating class, Alumni, and their friends, by the President of the Faculty.

---

Rush Medical College.—The Public Commencement exercises of Rush Medical College will be held on Wednesday evening, February 5th, in the lecture-room of the College, corner of North Dearborn and Indiana Streets. Although the time will probably have passed before this number of the Examiner reaches its readers, we will add that the degrees will be conferred by the President of the College, Prof. Blaney, and the valedictory addeess delivered by Prof. R. L. Rea.

---

State Medical Societies.—We are glad to see that the profession in some of our adjoining States have been reviving and reänimating their State Medical Societies. That of Missouri was recently started anew, in a manner to indicate a good prospect for permanency and usefulness. That of Wisconsin, met a few duys since, at Madison, and was attended by a goodly number of members. Our old friend Dr. H. Van Duzen, of Mineral Point, was chosen President; and we are quite sure that, under his administration, it will prosper.

PERSONAL.—Prof. W. H. Byford, of this city, sailed for Europe early in January. He intends to be absent six or eight months. His object is recreation and professional improvement. His place is being well filled in the Chicago Medical College, temporarily, by Dr. E. O. F. Roler.

GIVE AN INCH AND AN ELL IS TAKEN.—This old saying is capable of very extensive application in the ordinary affairs of life. It was strikingly illustrated in the advertising recently of two physicians, of good standing hitherto, who had determined to devote themselves exclusively to the treatment of syphilitic diseases. They called on several of the leading practitioners of the city, representing that they were engaging in this specialty in the same manner and as strictly within the rules of the profession as others do in the special department of ophthalmology. Under such representations, they obtained the consent of several prominent men to have their names used as references on an ordinary business card. Among those thus assenting, were the names of prominent members of both the medical college faculties in this city. In a few days, these gentlemen found their names not only appended to a simple business card, but that card published in the "Medical Column" of several of our city papers; in thousands of circulars; and, we are told, in several country newspapers. As soon as this extensive advertising business was discovered, those members of the faculty of the Chicago Medical College whose names were involved, immediately forbid the further publication of them in any form. Our own rule has been, for several years, to allow no use of our name for any *special* purpose whatever. It is the only safe rule.

TO PREVENT METALS FROM RUSTING.—Dip the article into very dilute nitric acid, and afterwards immerse it in linseed oil, allowing the superfluous portions to drain off. When the coating of oil is thoroughly dry, the article will be ready for use, and, thus protected, will remain bright for years.

MONEY RECEIPTS TO FEB. 1ST.—Drs. N. Senn, $3; J. B. Corr, 3; A. C. Corr, 3; J. W. Johns, 3; J. W. Bennett, 3; A. Patterson, 3; J. Priestman, 4; Q. M. Triplett, 3; J. H. Judson, 3; John D. Brundage, 3; V. C. Price, 3; W. L. Kreider, 3; F. Sikora, 3; J. A. Adrian, 3; J. R. Flood, 3; J. S. Bullock, 2; Chas. W. Oleson, 3; T. D. Palmer, 3; Geo. W. Crossley, 3; C. B. Reed, 3; J. P. Johnston, 3; L. H. Kennedy, 3; R. W. Winton, 3; N. W. Abbott, 5; J. F. Young, 3; W. H. Buchtel, 3; W. R. Fox, 6; John A. Macdonald, 4; J. S. Lawrence, 2; Hiram Nance, 3; Sumner Clark, 3; J. McLaughlin, 3; D. M. Bond, 3; Wm. Dillon, 3; James M. Kielly, 3; Geo. H. Calkins, 3; J. H. Newland, 3; A. Hager, 3; S. Rex Bucher, 3; D. Scott, 3; C. M. Robertson, 3.

INCOMPATIBILITY OF POT. IODID. AND POTASS. CHLORAT.—This is an important point in practice, for in syphilis, to act at the same time upon the ulceration of the mouth and the general malady, chlorat. potass. and pot. iodid. are frequently given. This practice is dangerous, as has been demonstrated by M. Vée; for the chlorate of potash, absorbed simultaneously with the iodide of potassium, may part with its oxygen, and transform it into the iodate, a poisonous agent. The recent experience of M. Melsens proves the possibility of this transformation.

This ought to suffice to prevent, were it only as a precautionary measure, the simultaneous administration of the chlorate of potash and the iodide of potassium.—*Gaz. Med. de Paris.*

MORTALITY AMONG PHYSICIANS AT GALVESTON, TEXAS.—Not less than eight practising physicians have died of yellow fever in Houston, Texas, during the prevalence of the epidemic.

### CHLORODYNE.

*To the Editor of the Medical Record*, SIR:—In the late edition of Aitken's Practice of Medicine (1866), Chlorodyne is several times recommended as a valuable remedy, and a formula is given on p. 471, vol i, which is said to be "a very useful one."

It is strange that a compound of this description should have obtained the sanction of physicians in the absence of any authentic or officinal directions for its preparation. In prescribing it, unless the nostrum is specified, the practitioner may not know which of many compounds will be furnished. I have before me five different formulas for chlorodyne, all differing in respect to ingredients and their proportions; and not one of them would furnish a good imitation of the English nostrum.

If a chloroform-anodyne is deemed worthy of the place it now holds in public estimation, it should have a uniform composition, or be made officinal, in order to prevent the hazard and uncertainty which must now be incident to its administration. The following is Dr. Aitken's formula:

℞. Chloroform f. ℥iv; Æth. Sulph. f. ℥ij; Theraicæ (Treacle) f. ℥j; Mucilag. Acaciæ f. ℥ij; Morph. Muriat. gr. viij; Acid. Hydrocyanic. dil. (2 per cent) ℥ij; Ol. Menth. Pip. M. iv ad vi. *Misce bene.*

Further directions are given about combining the materials, which we have attempted to follow, but, on standing, the whole of the ether and chloroform separate from the "constituent," so that the formula cannot be considered an eligible one.

About two years ago, the writer obtained a sample of Dr. Browne's genuine chlorodyne for examination. It was of a dark color, with an odor of chloroform and ether. By repeated and careful tests we failed to discover in it either cannabis or hydrocyanic acid. (The same result was obtained by Mr. Chas. Bullock, in his examination of a similar article.) The "constituent" was found to be molasses, gum acacia, and ext. glycyrrhiza. It contained no peppermint. There might possibly have been a small quantity of capsicum, but the pungent effects of this and the chloroform are so similar, that its presence may be considered doubtful. An imitation, containing only chloroform, morphia, and ether, resembles "the genuine" in sensible properties and operative effects. I published, accordingly, the following formula for chlorodyne, and should not have thought it of sufficient consequence to refer to it again, but for the notice taken of the compound in so respectable a work as Aitken's Practice of Medicine. The following is an imitation of Dr. J. Collis Browne's Chlorodyne:

Heat molasses in a water-bath, and remove the scum until it becomes clear. Mix one part of officinal mucilage of acacia with two parts of the molasses, to form the constituent.

Dissolve eight gr. sulphate of morphia in one drachm and a-half of water, by heat, and add eight gr. powdered extract of liquorice. To this solution, add one fluid drachm of conc. sulphuric ether. Add a portion of the constituent, and two fluid drachms of chloroform, and shake the mixture, then add enough of the constituent to make one fluid ounce.

Ten minims, or twenty drops, contain $\frac{1}{6}$ gr. of the morphia salt, two minims and a-half or ten drops of chloroform, and half the quantity of ether.

Chlorodyne thus prepared resembles the nostrum in pungency, color, density, and other properties. If the prescriber wishes to administer cannabis or hydrocyanic acid, they may be added extemporaneously. Truly yours,

*Rochester, N. Y., Dec., 1867.* W. W. ELY, M.D.

---

The question is so commonly raised as to the necessity of revaccination, and as to what is the best and surest method of vaccinating, that we think the following extracts from the report of the revaccination of the Prussian army for 1866, (taken from the *Berlin Klin. Wochens,*) will be of interest. The number vaccinated was 42,269; 86 per cent had plain scars, 9 per cent indistinct scars, and 5 per cent no marks of former vaccinations. The result of the vaccination was perfect in 60 per cent, imperfect in 13 per cent, nil in 27 per cent. Of these 27 per cent, about 30 per cent, or about 8 per cent of the whole number, on a second trial had perfect sores, making 68 per cent of perfect revaccinations. From the experiments with different methods of vaccination, the following conclusions are drawn:—1st. For military purposes, vaccination from arm to arm is to be preferred. 2d. The result of vaccination with glycerine lymph is generally more uncertain in proportion as the dilution of the lymph with glycerine is greater. 3d. Mixtures of one part lymph to eight, nine, or ten of glycerine are not to be depended upon in the revaccination of recruits. 4th. A less dilution produces in recruits good, genuine, and numerous pocks,and is, therefore, to be recommended for further exper. iments in the annual vaccinations.

---

Resections.—M. Sedillot has written a letter to the Imperial Society of Surgery, on the regeneration of bone. It is too long for quotation entire, but of great interest. He contradicts the two principles of sub-periosteal resection in the following terms:

One, to which Larghi has given the general name of sub-periosteal resection, is founded on the idea that the periosteum detached and isolated in the condition of a sheath or a flap, is able to renew or reproduce the subjacent bone from which it is stripped or raised.

The other principle, which I have called sub-periosteal gouging or scraping, has for its principle, that it is the periosteum, *only when attached* to the bone, that is able to renew it; and that, in consequence, the bone beneath the periosteum should be husbanded and preserved with the very greatest care.

After invoking the aid of the Baconian method of research, M. Sedillot quotes numerous series of experiments which, in his opinion, are sufficient to prove that the periosteal flaps, if completely isolated from the subjacent bone, are unable to reproduce it; "and that the so-called method of sub-periosteal resection is founded on a deplorable illusion;" and gives his opinion that the chief use of preserving the periosteum at all, is to supply a mould for the bony matter which is left in his method of periosteal gouging, and which really reproduces the bone.—*Ed. Med. Jour.*, *Gaz. des Hopitaux*, *Am. Jour. Med. Sciences.*

---

Spiders.—Father Babaz has communicated to the French Academy of Science an account of his experiments with spiders, and the manner in which he ascertained a fact hitherto unknown to naturalists, *viz.*, that most spiders possess not only the faculty of spinning a thread, but also that of projecting one or several, sometimes of the length of five or six metres, which they use to traverse distances with, and affix their thread to a given point for the support of their web. They even seem to have the power of directing the extremity of an ejaculated thread to a given point; they seem to feel for the place where it is most desirable to fix it. Certain spiders, the Thomisa Bufo, for instance, will eject a bunch of threads which, curling up in the air and shining in the sun with various hues, gives the insect the appearance of a peacock displaying his tail. But this is not all: spiders can fly and swim in the air, though they are heavier than alcohol. To perform this feat, they turn their back to the ground, and keep their legs closely folded up on the body, and in this posture sail about with perfect ease.

---

Professor Pajot of Paris proposes the following method of fixing the head in certain cases of embryotomy:

"I perforate the cranium in the usual manner, and introduce into the opening a little stick four or five centimetres long and as big as the little finger, to the middle of which a string has already been fastened. I introduce this stick endwise within the cranium, by means of a tamponning forceps. When it is completely introduced, I draw upon the string; the stick becomes horizontal, either before or behind, on one side or the other of the pelvis, according as I judge to be desirable. I have then the head entirely at my control. Entrusting the string to an assistant, I apply the instrument which I have chosen, cephalotribe or forceps, without being embarassed by the string, which takes up no room in the vagina, and I have thus remedied the mobility of the head, a cause giving great difficulty in certain operations, and especially I have remedied it without a new instrument. This, in a word, is my procedure."

---

The Missouri Medical Association was duly resuscitated on the 12th December, after a long sleep of ten years, and has inaugurated some wise measures relating to the future prosperity of medicine in the State. A communication from the St. Louis Medical Society, asking of the Legislature a Board of Medical Examiners to be appointed by the Missouri Association, with power to grant licenses, was adopted, and a committee appointed to prepare a suitable memorial. They also endorsed most emphatically the stand taken in the matter of medical education by the recent convention of Medical Teachers.

---

Detecting Belladonna.—Mr. H. C. Scorby read a paper lately before the Sheffield Literary and Philosophical Society, in which he set forth the difficulties which the toxicologist encounters in his efforts to prove a case of poisoning by belladonna. These difficulties are obviated by the use of the micro-spectroscope. The spectrum of the juice of belladonna is very distinct, especially when the coloring matter has been added to a solution of carbonate of soda. A small fraction of a single berry is sufficient to produce the spectrum bands characteristic of the belladonna.

---

The Chair in the French Academy of Sciences made vacant by the death of Civiale, has been filled by the election of Baron Larrey, who received forty-five of the fifty-six votes cast.

---

Dr. D. S. Young has been appointed to the Chair of Surgery in the Cincinnati Medical College.—*Medical Record.*

Treatment of Stricture of the Urethra by Immediate Dilatation.—Mr. Holt continues to be highly successful in the use of his stricture dilator. He records in the *Lancet* 114 cases occurring in private and hospital practice, during the past year, upon which he operated. Not a single bad result followed, and only one complication—a small abscess of the penis—resulting from breaking the instrument. Many of his cases have been of the most unpromising character, and one especially, that of an India officer, deserves mention. He had abscess of the prostate, following stricture and infiltration of urine. The abscess broke into the rectum, establishing recto-vesical fistula, and there were fistulous openings in the perineum and right buttock. But little urine escapes through the penis, the patient being obliged to undress and sit upon a vessel when he desired to urinate. The suffering and impairment of health were great. No instrument was passed at the first trial; but at the second, the patient being under the influence of chloroform, the dilator was passed and the stricture split with a No. 12 tube. A gum-elastic catheter was retained in the urethra. The patient never had a bad symptom. The fistulous openings rapidly healed, and he was soon able to micturate with perfect ease.—*Pacific Med. & Surg. Journal.*

---

### AMERICAN MEDICAL ASSOCIATION—PRIZE ESSAYS FOR 1868.

The American Medical Association offers two prizes of *One Hundred Dollars* each, for the best two original essays upon subjects of professional interest; the Committee reserving the right to reject all, unless deemed fully worthy.

Competitors for these prizes must forward their essays to Dr. Charles Woodward, Cincinnati, Ohio, free of expense, on or before the 1st of April, 1868.

Each essay must be accompanied by a sealed note containing the author's name and address, and on this sealed packet must be inscribed some sentiment, motto, or device, corresponding to a like sentiment, motto, or device on the essay.

Charles Woodward, Chairman,

W. W. Dawson,

E. B. Stevens,

Roberts Bartholow,

P. S. Connor,

} Committee.

Medical journals throughout the country are requested to copy.

Mortality of Chicago for 1867:—We copy the following Report from the proceedings of the Board of Health, as published in the *Chicago Daily Times*. It contains much that may be useful for future reference:—[Ed.]

The Health Department of Chicago constitutes a branch of the municipal government which has in charge the most important interests of the citizen. The eradication of disease and the adoption of precautionary measures to restrain the spread of diseases and epidemics, is a matter so important that all persons are more or less interested in the action of those who have these important objects in charge.

Since last July, the Health Department has endeavored to systematize all its workings and reduce everything pertaining to sanitary benefits to a method. Rigorous registration and efficient executive officers have effected this, and in the limited mortality of the past year is found the best illustration of what can be done by an organization devoted to the sanitary improvement of the City.

THE WORK OF THE YEAR.

The following tabular statement of the work done by the department during the year exhibits in detail what has been done to conserve the public health:—

NUMBER OF NUISANCES REPORTED AND ABATED UPON NOTICE.

| | | | |
|---|---|---|---|
| Ashes and rubbish on streets, and yards, and alleys | 1492 | Filthy hog-pens | 79 |
| Garbage | 1336 | Filthy hide stores and cellars | 45 |
| Manure piles | 9757 | Filthy privies | 1775 |
| Full privies | 8070 | Filthy packing-houses | 7 |
| Filthy areas and alleys | 5634 | Filthy soap factories | 3 |
| Filthy butcher-shops | 6 | Filthy stables | 1500 |
| Filthy cellars | 125 | Filthy sinks | 900 |
| Filthy cess-pools and cisterns | 149 | Filthy slaughter-pens | 4 |
| Filthy drains | 580 | Filthy tanneries | 5 |
| Filthy distilleries | 4 | Filthy vacant lots | 309 |
| Filthy gutters on st's and alleys | 1489 | Filthy water closets | 513 |
| Filthy grocery stores | 10 | Total | 34,139 |
| Filthy houses | 349 | | |

| | |
|---|---|
| No. of notices served to connect with street sewers (Premisis connected 1097.) | 1857 |
| New privy vaults built | 831 |
| Total Number of all notices served | 36,827 |
| Total Number of all notices served in 1866 | 16,787 |
| Loads of swill removed by City scavengers | 6,842 |
| Loads of ashes removed by City scavengers | 10,360 |
| Total | 17,202 |

| | |
|---|---|
| Barrels of rotten apples removed | 256 |
| Barrels of rotten potatoes removed | 389 |
| Barrels of rotten eggs removed | 45 |
| Barrels of rotten fish removed | 63 |
| Barrels of rotten pickels removed | 500 |
| Total | 1,253 |
| Baskets and boxes of rotten peaches removed | 459 |
| Baskets and boxes of rotten grapes removed | 45 |
| Baskets and boxes of rotten oranges and lemons removed | 257 |
| Total | 761 |
| Barrels and bags of rotten poultry removed | 35 |
| Number of dead animals removed:— | |
| Dead horses removed | 147 |
| Dead sheep removed | 67 |
| Dead cows removed | 34 |
| Dead hogs removed | 56 |
| Dead dogs and cats removed | 11,251 |
| Dead chickens removed | 1,850 |
| Total | 13,405 |
| Main sewers disinfected | 3 |
| Private sewers disinfected | 1,735 |
| Sloughs and slips | 35 |
| Total | 1,773 |
| Miles of streets and alleys disinfected | 146 |
| Number of houses disinfected | 79 |
| Number of privies disinfected | 2150 |
| Number of small-pox cases reported | 722 |
| Number of varioloid cases reported | 244 |
| Total | 966 |

SMALL-POX.

| | |
|---|---|
| Number of patients removed to the small-pox hospital | 182 |
| Number of houses under sanitary inspection where patients resided | 784 |
| Number of summons served for violation of the health ordinances | 1138 |
| Fines assessed in the police court | $5133,20 |
| Twenty-eight persons were arrested for dumping manure on the streets, and the fines assessed upon them in the police court amounted to | 259,00 |
| Total | $5392,20 |

DISINFECTANTS.

Disinfectants and absorbents used:—

| | |
|---|---|
| Copperas, lbs. | 24,596 |
| Sal chloride zinc, bbls. | 2 |
| Carbonic acid, bbls. | 7 |
| Heavy Oil, bbls. | 32 |
| Disinfecting compounds, bbls. | 112 |
| Gypsum, bbls. | 105 |
| Lime, bbls. | 755 |

### MORTALITY FOR 1867.

The following is the mortality report for the year. Its details and comparisons will prove of interest to the public:—

| | |
|---|---|
| Abortion | 1 |
| Abscess | 9 |
| Accidents | 169 |
| Ædema glottidis | 1 |
| Anæmia | 3 |
| Anæsarca | 1 |
| Anginia | 5 |
| Aorta, aneurism of | 1 |
| Ascites | 1 |
| Asphyxia | 2 |
| Asthma | 3 |
| Apoplexy | 22 |
| Atrophy | 5 |
| Birth, premature | 79 |
| " still | 252 |
| Bowels, inflammation | 52 |
| " obstruction | 5 |
| Brain, congestion of | 48 |
| " disease of | 22 |
| " inflammation | 59 |
| Bright's disease | 5 |
| Bronchitis | 25 |
| Cancer | 22 |
| " of duodenum | 1 |
| " of face | 1 |
| " of intestines | 1 |
| " of liver | 2 |
| " of stomach | 7 |
| " of uterus | 6 |
| Canker sore mouth | 3 |
| Carries, knee | 1 |
| " spine | 1 |
| Catarrh | 2 |
| Cerebritis | 3 |
| Chest, disease of | 1 |
| Chlorosis | 1 |
| Chicken-pox | 2 |
| Cirrhosis | 1 |
| Cholera morbus | 19 |
| " infantum | 542 |
| Cold | 11 |
| Convulsions | 398 |
| Croup | 98 |
| Cyomosis | 6 |
| Cyanche tonsillaries | 1 |
| Debility | 74 |
| Delirium tremens | 14 |
| Diabetes | 1 |
| Diarrhœa | 144 |
| Diphtheria | 76 |
| " brain | 8 |
| " chest | 2 |
| " heart | 1 |
| Dropsy | 75 |
| Dysentery | 79 |
| Dyspepsia | 1 |
| Encephalites | 7 |
| Endrocardites | 2 |
| Enteritis | 29 |
| Enterocolites | 3 |
| Epilepsy | 13 |
| Erysipelas | 13 |
| Exhaustion | 1 |
| Exposure | 1 |
| Fever, bilious | 14 |
| " congestive | 24 |
| " puerperal | 21 |
| " putrid | 1 |
| " remittent | 9 |
| " scarlet | 99 |
| " spotted | 1 |
| " typhoid | 163 |
| " typhus | 27 |
| " intermittent | -- |
| Fungus hemadotes | 1 |
| Gall bladder, disease | 1 |
| Gangrene | 5 |
| Gastrites | 14 |
| Gastrœntrites | 2 |
| Gravel | 1 |
| Hæmatemesis | 2 |
| Hæmoptysis | 1 |
| Hemorrhage, umbilical | 1 |
| Heart, disease of | 59 |
| Hemiplegia | 1 |
| Hepatitis | 4 |
| Hernia | 4 |
| Hip disease | 1 |
| Hydrocephalus | 59 |
| Hypertrophy of heart | 2 |
| Insanity | 2 |
| Inanition | 28 |
| Intemperance | 2 |
| Jaundice | 6 |
| Kidneys, disease of | 7 |
| " inflammation | 1 |
| Laryngitis | 10 |
| Leg, commin'ted fract. | 1 |
| Liver, disease of | 3 |
| " corrhasis of | 2 |
| " inflammation of | 10 |
| Lungs, congestion of | 30 |
| " disease of | 27 |
| " hemorrhage of | 4 |
| Lupus | 1 |
| Measles | 87 |
| Meningitis | 38 |
| " cereb.-spinal | 19 |
| " tuberculous | 5 |
| Morbus coxarius | 2 |
| Mouth, malformation | 1 |
| Myelitis | 2 |
| Nephritis | 3 |
| Neuralgia | 1 |
| Old Age | 72 |
| Œsophagus, inflam'on | 1 |
| Ovarian disease | 2 |
| Over-exertion | 1 |
| Paralysis | 21 |
| Paraphlegia | 1 |
| Pericarditis | 2 |
| Peritonitis | 15 |
| Pharyngitis | 1 |
| Phlegmasia dolens | 2 |
| Phlebitis | 1 |
| Phthisis pulmonalisis | 398 |
| Pneumonia | 159 |
| Potts' disease | 1 |
| Porrporra | 1 |
| Pyemia | 1 |
| Rheumatism | 7 |
| Rubia | 1 |
| Scrofula | 7 |
| Small-pox | 115 |
| Softening spinal cord | 1 |
| Spine, disease of | 9 |
| Stomach, disease of | 4 |
| " congestion of | 1 |
| Stricture | 1 |
| Suicide | 19 |
| Sunstroke | 7 |
| Suffocation | 2 |
| Syphilis | 2 |
| Tabes mesenterica | 115 |
| Tapeworm | 1 |
| Teething | 148 |
| Tetanus | 3 |
| Tonsils, inflammation | 1 |
| Throat, disease of | 2 |
| " malformation | 1 |
| Thrush | 1 |
| Trismus | 1 |
| Tumor | 2 |
| Ulceration | 3 |
| " bowels | 1 |
| " stomach | 1 |
| Uræmia | 1 |
| Urine, suppression of | 1 |
| Uterus, rupture of | 1 |

| | | | | | |
|---|---|---|---|---|---|
| Vesical calculus | 1 | Worms | 2 | | —— |
| Whooping-cough | 62 | Unknown | 138 | Total | 4604 |

AGES.

| | | | | | |
|---|---|---|---|---|---|
| Under 5 | 2784 | 40 to 50 | 237 | 90 to 100 | 4 |
| 5 to 10 | 182 | 50 to 60 | 140 | 100 to 110 | 2 |
| 10 to 20 | 176 | 60 to 70 | 126 | Unknown | 97 |
| 20 to 30 | 381 | 70 to 80 | 61 | | —— |
| 30 to 40 | 378 | 80 to 90 | 36 | Total | 4604 |

NATIVITY.

| | | | | | |
|---|---|---|---|---|---|
| Chicago | 2460 | Holland | 27 | Russia | 1 |
| Other parts U. S. | 675 | Ireland | 428 | Scotland | 36 |
| Austria | 6 | Isle of Man | 1 | Shetland Islands | 1 |
| Belgium | 1 | Island of St. Helena | 1 | Sweden | 57 |
| Bohemia | 57 | Italy | 1 | Switzerland | 10 |
| Canada | 51 | Norway | 92 | Wales | 5 |
| Denmark | 12 | Nova Scotia | 2 | On the Sea | 3 |
| England | 97 | Prussia | 27 | Unknown | 34 |
| France | 14 | Poland | 3 | | —— |
| Germany | 482 | | | Total | 4604 |

Deaths from Still-birth and Accidental Causes—1866, 331; 1867, 521.

Deaths by Disease—1866, 5594; 1867, 4083; Decrease in 1867, 1511.

Interments at various Cemeteries in October, 1866, 1525; Deaths reported in October, 1866, 1170; Number not accounted for, 355.

DEATHS BY MONTHS IN 1867.

| | | | | | |
|---|---|---|---|---|---|
| January | 299 | June | 283 | October | 428 |
| February | 255 | July | 597 | November | 360 |
| March | 280 | August | 696 | December | 380 |
| April | 278 | September | 507 | | —— |
| May | 241 | | | Total | 4604 |

BIRTHS SINCE JULY 1, 1867.

| | | | | | |
|---|---|---|---|---|---|
| July | 412 | October | 459 | December | 508 |
| August | 481 | November | 452 | | —— |
| September | 497 | | | Total | 2809 |

Males 1409 | Females 1400

MORTALITY BY WARDS SINCE JULY 1, 1867.

| | | | | | |
|---|---|---|---|---|---|
| First Ward | 35 | Seventh Ward | 343 | Thirteenth Ward | 141 |
| Second Ward | 121 | Eighth Ward | 192 | Fourteenth Ward | 201 |
| Third Ward | 146 | Ninth Ward | 166 | Fifteenth Ward | 240 |
| Fourth Ward | 142 | Tenth Ward | 93 | Sixteenth Ward | 173 |
| Fifth Ward | 162 | Eleventh Ward | 191 | | —— |
| Sixth Ward | 202 | Twelfth Ward | 235 | Total | 2785 |

| | |
|---|---|
| Total deaths in 1866 | 5925 |
| Total deaths in 1867 | 4604 |
| Decrease | 1321 |

EXPENDITURES.

Amount expended since April 1, 1867:—

| | | | | | |
|---|---|---|---|---|---|
| Scavenger work | $28,344 | Small-pox Hospital | 2,500 | Salaries | 28,000 |
| Disinfectants | 1,500 | Cholera " (rep's) | 63 | Sundries | 745 |
| Stationery | 375 | Office furniture, etc. | 500 | | —— |
| Printing | 1,150 | | | Total | $63,177 |

